# The oral glucose tolerance test for the diagnosis of diabetes mellitus in patients during acute coronary syndrome hospitalization: a meta-analysis of diagnostic test accuracy

**DOI:** 10.1186/1475-2840-11-155

**Published:** 2012-12-27

**Authors:** Yicong Ye, Hongzhi Xie, Xiliang Zhao, Shuyang Zhang

**Affiliations:** 1 Department of Cardiology, Peking Union Medical College Hospital, Peking Union Medical College & Chinese Academy of Medical Sciences, Beijing, Dongcheng District, 100730, China

**Keywords:** Acute coronary syndrome, Oral glucose tolerance test, Accuracy, Meta-analysis

## Abstract

**Background:**

The appropriateness of the routine performance of an oral glucose tolerance test (OGTT) to screen for diabetes mellitus (DM) during acute coronary syndrome hospitalization is still under debate.

**Methods:**

A systematic search of databases (MEDLINE [1985 to March 2012], EMBASE [1985 to March 2012]) was conducted. All prospective cohort studies assessing the accuracy or reproducibility of an OGTT in ACS or non-ACS individuals were included. A bivariate model was used to calculate the pooled sensitivity (SEN), specificity (SPE), positive likelihood ratio (PLR), negative likelihood ratio (NLR), and diagnostic odds ratio (DOR). Heterogeneity was explored using subgroup analysis and meta-regression.

**Results:**

Fifteen studies with 8,027 participants were included (10 ACS and 5 non-ACS studies). The pooled results on SEN, SPE, PLR, NLR, and DOR were 0.70 (95% CI, 0.60-0.78), 0.91 (95% CI, 0.86-0.94), 7.6 (95% CI, 4.9-11.7), 0.33 (95% CI, 0.25-0.45), and 23 (95% CI, 12–41), respectively. The OGTT has a slightly lower SPE in diagnosing DM in ACS than in non-ACS patients (0.86 [95% CI 0.81-0.92] versus 0.95 [95% CI 0.93-0.98], *p*<0.01), while the SEN values are comparable (0.71 [95% CI 0.60-0.82] versus 0.67 [95% CI 0.54-0.81], *p*=0.43). After adjusting the interval between repeated tests and age, the meta-regression did not show a difference in DOR between ACS and non-ACS studies.

**Conclusions:**

Despite the discrepancy in the interval between the two OGTTs, performing an OGTT in patients with ACS provides accuracy that is similar to that in in non-ACS patients. It is reasonable to screen patients hospitalized for ACS for previously undiagnosed DM using an OGTT.

## Introduction

Numerous studies have demonstrated that hyperglycemia is common among patients with acute coronary syndrome (ACS) [1,2], and the relationship between hyperglycemia and increased mortality risk in ACS has been well established across various glucose metrics [3,4].

However, considering its accuracy and reproducibility in stress condition, the routine performance of an oral glucose tolerance test (OGTT) to diagnose diabetes during the acute phase of ACS is still the subject of ongoing debate. The European guidelines on diabetes, pre-diabetes, and cardiovascular diseases recommend the performance of an OGTT in patients with established cardiovascular disease [5]. Furthermore, the European guidelines on the management of acute myocardial infarction in patients presenting with persistent ST-segment elevation specify that an OGTT should be performed before or shortly after hospital discharge [6]. In regards to the management of hyperglycemia in ACS, the NICE (National Institute for Health and Clinical Excellence) does not recommend the routine use of the OGTT in patients with hyperglycemia after ACS without known diabetes if hemoglobin A1C and fasting blood glucose levels are within the normal range [7]. A scientific statement from the American Heart Association Diabetes Committee of the Council on Nutrition, Physical Activity, and Metabolism does not encourage routine use of the OGTT for screening during the hospital stay [8].

Thus, this meta-analysis of prospective cohort studies to determine the accuracy of the OGTT in the diagnosis of diabetes in the acute phase of ACS compared to that in non-ACS cases was conducted to clarify this dispute with evidence.

## Methods

A protocol was designed that detailed the objective of our analysis, the criteria for study inclusion/exclusion, the assessment of study quality, the primary outcome, and the statistical methods in accordance with the MOOSE guideline for meta-analysis of observational studies [9].

### Data sources and searches

A search of MEDLINE (1985 to March 2012) and EMBASE (1985 to March 2012) via EMBASE.com was conducted to identify all studies involving diagnostic tests assessing the value of the OGTT in the diagnosis of diabetes mellitus in subjects with or without ACS (Additional file 1) (Yicong Ye and Hongzhi Xie). In addition, a manual search of the literature using the references of original manuscripts, reviews, and meta-analyses was performed. Finally, a search of the Cochrane database of ongoing systemic reviews was conducted. No language restriction was imposed.

### Study selection

The study eligibility was independently determined by two reviewers (Yicong Ye and Hongzhi Xie). Disagreement was resolved by consensus. The study eligibility criteria included: 1) published prospective cohort studies, 2) performing the first OGTT during ACS hospitalization (only for ACS studies), 3) repeating an OGTT more than 1 week and less than 3 years after the first one, and 4) use of a 75 g OGTT.

The following were criteria for exclusion: 1) pregnant and pediatric individuals, 2) patients with other chronic diseases, other than coronary heart disease, such as cystic fibrosis, polycystic ovary syndrome, acromegaly, liver disease, and renal disease, 3) studies in which the OGTT was repeated only in subjects with abnormal or normal OGTT results in the first test, and 4) studies with intended medical intervention (life-style change or medication) between administration of the two OGTTs.

Attempts were made to contact the author for further information on studies that fulfilled the above criteria but did not have sufficient data to build a two-by-two table before they were excluded from the final analysis.

### Data extraction and quality assessment

Data extraction was carried out independently by two authors (Yicong Ye and Hongzhi Xie). Disagreements were resolved by discussion between the two reviewing authors. From each included trial, information was extracted on: 1) the study population, 2) the published language, 3) the time interval between administration of the two OGTTs, 4) the reference standard of diabetes, 5) mean age of study population and 6) the true positive value, false positive value, false negative value, and true negative value of each included study.

Study quality was assessed using the QUADAS list, with each item scored as “yes”, “no”, or “unclear” [10]. The results are presented in the text and in a graph. A summary score was not calculated when estimating the overall quality of an article since the interpretation of such summary scores is problematic and potentially misleading. The items in the QUADAS tool and their interpretation are presented in Additional file 1.

### Data synthesis and analysis

The WHO (World Health Organization) 1985 [11], ADA (American Diabetes Association) 2003 [12], or WHO 1999 [13] criteria was used as the reference standard depending on which criteria had been used in each study. All the included patients can be classified into two groups: diabetes and non- diabetes. The diagnostic threshold was the same as the reference standard in each included study. Accordingly, the two-by-two tables were constructed. The data in the two-by-two tables were used to calculate the sensitivity and specificity for each study. The individual study results were presented graphically by plotting the estimates of sensitivity (SEN) and specificity (SPE), and their 95% confidence intervals (95% CI), in paired forest plots.

A bivariate model was used for the meta-analysis of the pairs of SEN and SPE and for the construction of a summary receiver operating characteristic (ROC) curve [14]. This summary ROC curve represents the change in diagnostic accuracy according to changes in the cutoff value. The bivariate random effects approach enabled the calculation of summary estimates of SEN, SPE, the positive likelihood ratio (PLR), the negative likelihood ratio (NLR), and the diagnostic odds ratio (DOR) while correctly dealing with the different sources of variation: (1) the imprecision by which SEN and SPE were measured within each study, (2) variation beyond chance in SEN and SPE between studies, and (3) any correlation that might exist between SEN and SPE.

The heterogeneity (or absence of homogeneity) of the results between studies was assessed statistically using the quantity *I*^*2*^, which describes the percentage of total variation across studies that is attributable to heterogeneity rather than chance [15]. Covariates were incorporated in the bivariate model to examine the effect of potential sources of heterogeneity across subgroups of studies. Due to the bivariate nature of the model, the effects of covariates on SEN and SPE can be modeled separately. The following covariates were analyzed to explore the heterogeneity: 1) Study population: the major objective of the studies was to compare the diagnostic accuracy in patients with and without ACS; 2) Threshold: although different criteria were used in the studies (including WHO 1985, ADA 2003, and WHO 1999), all studies used the same cutoff value of a 2-hour OGTT (11.1mmol/L) to diagnose diabetes. The major difference is that some of the studies included FBG in the diagnosis criteria, while others did not. Thus, the studies were divided into 2 subgroups (with or without FBG). 3) Blood sample: different reproducibility values for plasma and capillary glucose have been reported. 4) Interval between repeated tests (within 2 months versus more than 2 months): a prolonged interval between two OGTTs may also lead to poor reproducibility even though the optimal interval is still unknown. 5) Age: aging is associated with degradation of the pancreatic β-cells which may affect the accuracy of OGTT.

Meta-regression was conducted to further explore the heterogeneity quantitatively among the studies and to determine the diagnostic accuracy of the OGTT in different conditions. The lnDOR was used as the dependent variable. The standard error of the lnDOR was used to measure the within-study variability, and the residual maximum likelihood method to estimate the between-study variance. Factors associated with significantly different SEN and/or SPE in the subgroup analysis were included in the meta-regression model as covariates.

The potential publication bias was assessed using the Deeks funnel plots [16]. The studies by Eschwege et al. and De Vegt F et al. had much larger sample sizes and much longer intervals between the administrations of the two OGTTs, compared with the other studies included in the meta-analysis (Table 1). In order to assess the effect of this study on the pooled result, a post-hoc sensitive analysis was performed to calculate DOR for ACS in the meta-regression model without the studies by Eschwege et al. and De Vegt F et al. In addition, the diagnostic criteria for DM have changed over time, thus the criteria used in the included studies has varied. Another post-hoc sensitive analysis was performed in order to calculate DOR for ACS in the meta-regression model without the study using the 1985 WHO criteria.

**Table 1:** Characteristics of the studies included in the meta-analysis

**Studies**	**Study population**	**Language**	**Reference standard**	**Interval between repeated tests**	**Age**	**Sample**	**Sample size**	**Diabetes**			
								**TP**^**‡‡**^	**FP**	**FN**	**TN**
Wei et al. 2011	ACS^*^	English	WHO^¶^1999 (2hOGTT^**^+FBG^††^)	>1 week	65.6	Plasma	94	23	4	0	67
Ilany et al. 2011	ACS	English	Unknown (2hOGTT+FBG)	3-24 months	57	Plasma	29	0	10	1	18
Bronisz et al. 2011	STEMI^†^	English	WHO1999 (2hOGTT)	3 months	56.5	Plasma	200	8	20	2	170
Jimenez-Navarro et al. 2010	PCI^‡^ (ACS 80%)	English	WHO1999 (2hOGTT)	1 months	60.8	Capillary	88	9	21	7	51
Lewczuk et al. 2009	ACS	Polish	Unknown (2hOGTT+FBG)	12 months	51.9	Plasma	69	14	3	5	47
Knudsen et al. 2009	STEMI	English	WHO1999 (2hOGTT+FBG)	3 months	58	Plasma	201	5	17	5	174
Srinivas-Shankar et al. 2008	NSTEMI	English	WHO1999 (2hOGTT+FBG)	3 months	65	Plasma	49	3	3	2	41
Lankisch et al. 2008	AMI^§^	English	WHO1999 (2hOGTT+FBG)	3 months	62.7	Plasma	62	11	8	8	35
Choi et al. 2005	AMI	English	WHO1999 (2hOGTT+FBG)	3 months	60.1	Plasma	30	7	3	2	18
Tenerz et al. 2003	AMI	English	WHO1999 (2hOGTT+FBG)	3 months	63.2	Capillary	142	25	22	10	85
Liu et al. 2007	General population	Chinese	ADA^#^2003 (2hOGTT+FBG)	2-3 weeks	NA	Plasma	259	30	17	8	204
Eschwege et al. 2001	General population	English	WHO1999 (2hOGTT+FBG)	30 months	50	Plasma	5400	105	102	171	5022
De Vegt et al. 2000	General population	English	WHO1985 (2hOGTT+FBG)	2-6 weeks	61.7	Plasma	1109	85	25	31	968
Ko et al. 1998	General population	English	WHO1985 (2hOGTT+FBG)	6 weeks	41	Plasma	212	24	17	56	115
Farrer et al. 1995	3 months post-elective CABG^||^	English	WHO1985 (2hOGTT+FBG)	10 days	56.8	Plasma	81	9	5	3	64

^*^Acute coronary syndrome; ^†^ST-elevation myocardial infarction; ^‡^Percutaneous coronary intervention; ^§^Acute myocardial infarction;

^||^Coronary artery bypass graft; ^¶^World Health Organization; ^#^American Diabetes Association; ^**^Oral glucose tolerance test;

^††^Fasting blood glucose; ^‡‡^TP: True positive; FP: False positive; FN: False negative; TN: True negative**.**

All analyses were performed using STATA version 11.0 (Stata Corp; College Station, TX). All statistical tests were two-sided, with a *p* value of 0.05 denoting statistical significance.

## Results

### Characteristics and methodological quality of included studies

**Figure 1:**
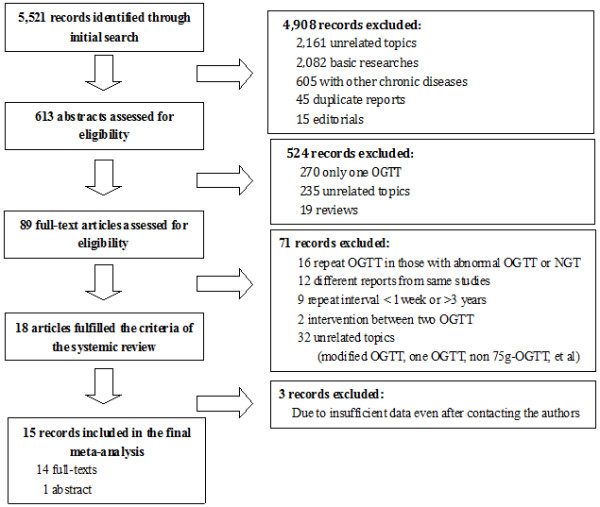
**Flowchart of study selection.** NGT: normal glucose tolerance. OGTT: oral glucose tolerance test.

**Figure 2:**
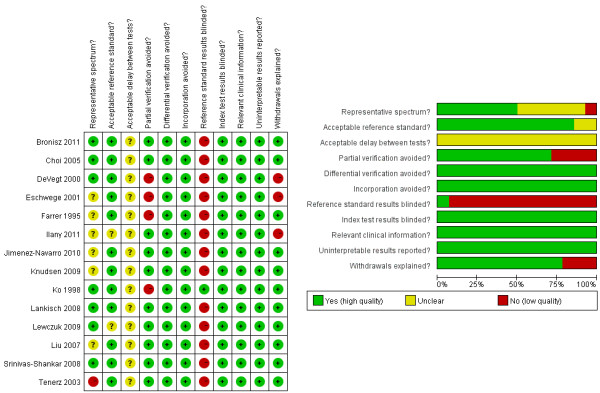
Risk of bias.

Of the 5,521 references identified in the initial search, only 18 reports fulfilled the criteria for inclusion in the meta-analysis. After communicating with the authors, 15 reports offered sufficient data to build a two-by-two table and thus were included in the final meta-analysis (Figure 1) [17-31]. Of the 15 included studies involving 8,027 subjects, 10 studies included patients with ACS, and the remaining consisted of non-ACS individuals. The characteristics of the included studies are detailed in Table 1. The results on the methodological quality of the included studies are presented in text form in Additional file 1 and in a graph in Figure 2.

### Pooled results and hierarchic summary ROC curve

**Figure 3:**
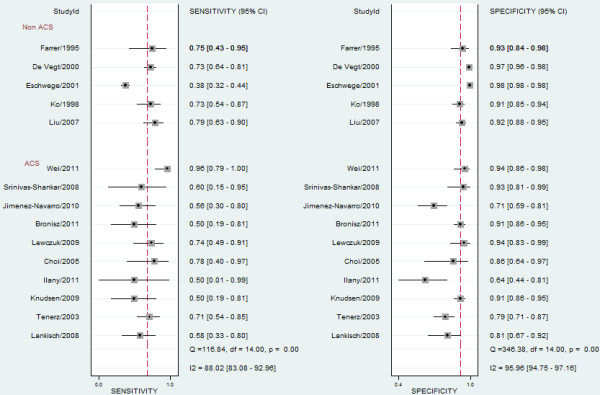
**Paired forest plots of sensitivity and specificity.** ACS: acute coronary syndrome.

**Figure 4:**
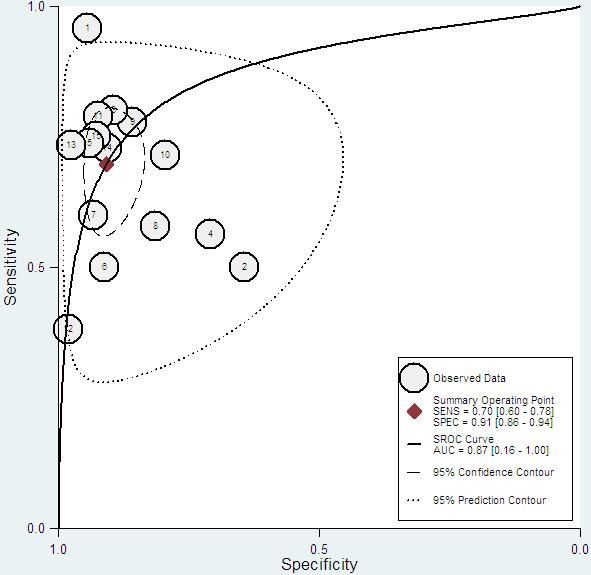
**Hierarchical summary receiver operating characteristic (SROC) curve.** AUC: area under curve.

The SEN of the included studies ranged from 0.38 to 0.96, whereas the SPE ranged from 0.64 to 0.98 (forest plots in Figure 3). The hierarchical summary ROC curve represents the relationship between sensitivity and specificity across the included studies with a 95% confidence ellipse and a 95% prediction ellipse (Figure 4). The area under the summary ROC curve (AUC) was 0.87 (95% CI, 0.16-1.00). Using the bivariate model, the pooled results for SEN, SPE, PLR, NLR, and DOR were 0.70 (95% CI, 0.60-0.78), 0.91 (95% CI, 0.86-0.94), 7.6 (95% CI, 4.9-11.7), 0.33 (95% CI, 0.25-0.45), and 23 (95% CI, 12–41), respectively. The *I*^2^ value of all measures was 99% (95% CI, 98-99%), indicating significant heterogeneity across the included studies.

### Subgroup analysis, meta-regression and publication bias

**Figure 5:**
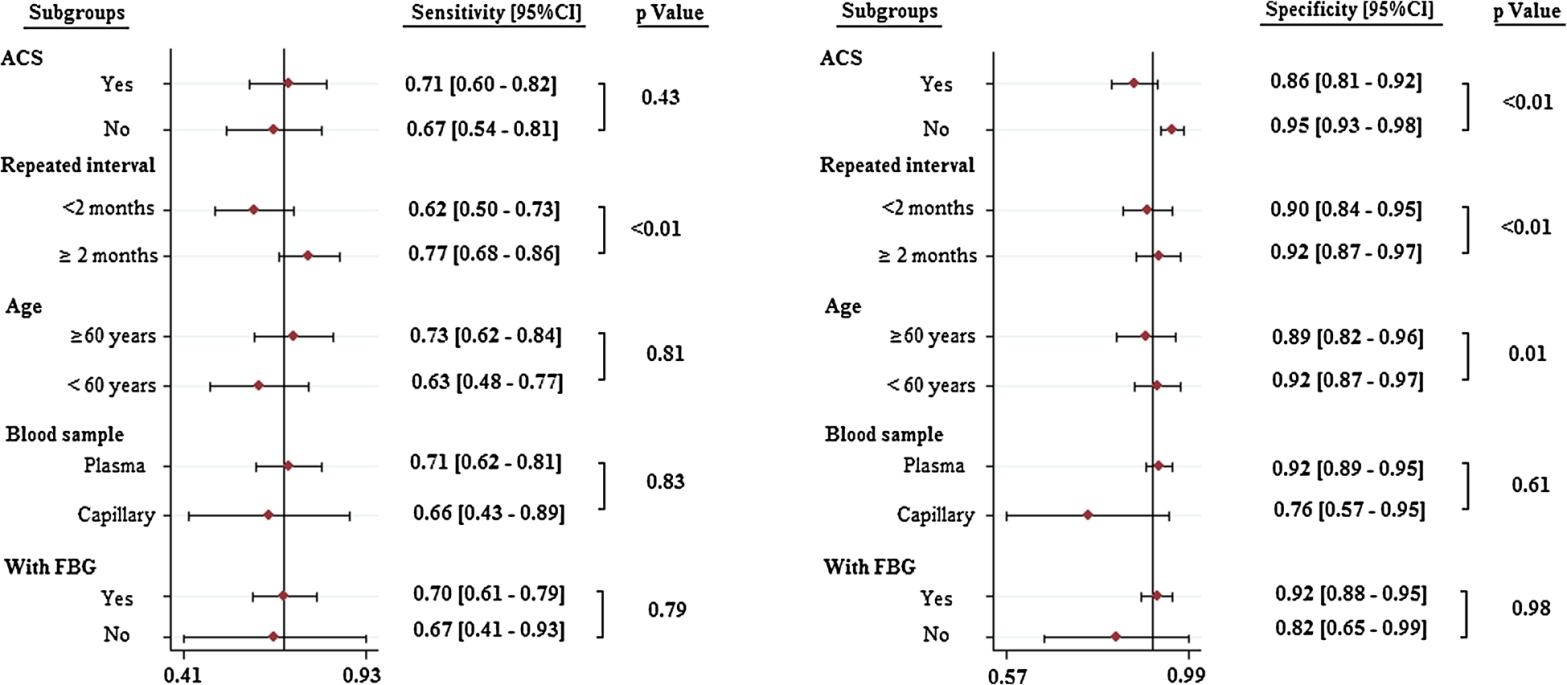
**Subgroup analysis (sensitivity and specificity).** ACS: acute coronary syndrome FBG: fasting blood glucose.

The subgroup analyses demonstrated that the OGTT performed in ACS patients has similar SEN (0.71 [95%CI, 0.60-0.82] versus 0.67 [95% CI, 0.54-0.81], *p*=0.43) but a slightly lower SPE (0.86 [95% CI, 0.81-0.92] versus 0.95 [95% CI, 0.93-0.98], *p*<0.01) compared with non-ACS patients. A prolonged interval between repeated tests (more than 2 months) is also associated with lower SEN (0.62 [95% CI, 0.50-0.73] versus 0.77 [95% CI, 0.68-0.86], *p*<0.01) and SPE (0.90 [95% CI, 0.84-0.95] versus 0.92 [95% CI, 0.87-0.97], *p*<0.01). Compared with the younger age (< 60 years) group, advanced age (≥60 years) is associated with lower SPE (0.89 [95% CI, 0.82-0.96] versus 0.92 [95% CI, 0.87-0.97], *p*=0.01) while the SEN is similar (0.73 [95% CI, 0.62-0.84] versus 0.63 [96% CI, 0.48-0.77], p=0.81). However, using a different threshold (2-hour OGTT with or without FBG) or blood sample (plasma glucose or capillary glucose) did not lead to different diagnostic accuracy (all *p*>0.05) (Figure 5).

Since ACS, interval between repeated tests and age were found to be associated with different SEN and/or SPE in the subgroup analysis, multiple meta-regressions were performed to further determine the effect of these factors on the DOR. However, none of these covariants was found to be associated with different diagnostic accuracy in the multiple meta-regression model (Table 2).

**Table 2:** Results of multiple meta-regression

**Variants**	**DOR**	**95% CI for DOR**	***p *value**
Acute coronary syndrome	0.27	0.03-2.33	0.21
Interval between repeated tests	0.79	0.13-4.75	0.78
Age	1.04	0.91-1.19	0.51

Tau^2^ (estimate of between-study variance) = 0.89.

Residual I^2^ (percentage of residual variation due to heterogeneity) = 63.49%.

Adjusted R^2^ (proportion of between-study variance explained) = 12.10%.

Model F = 1.10, P = 0.39.

*Diagnostic odds ratio.

**Figure 6:**
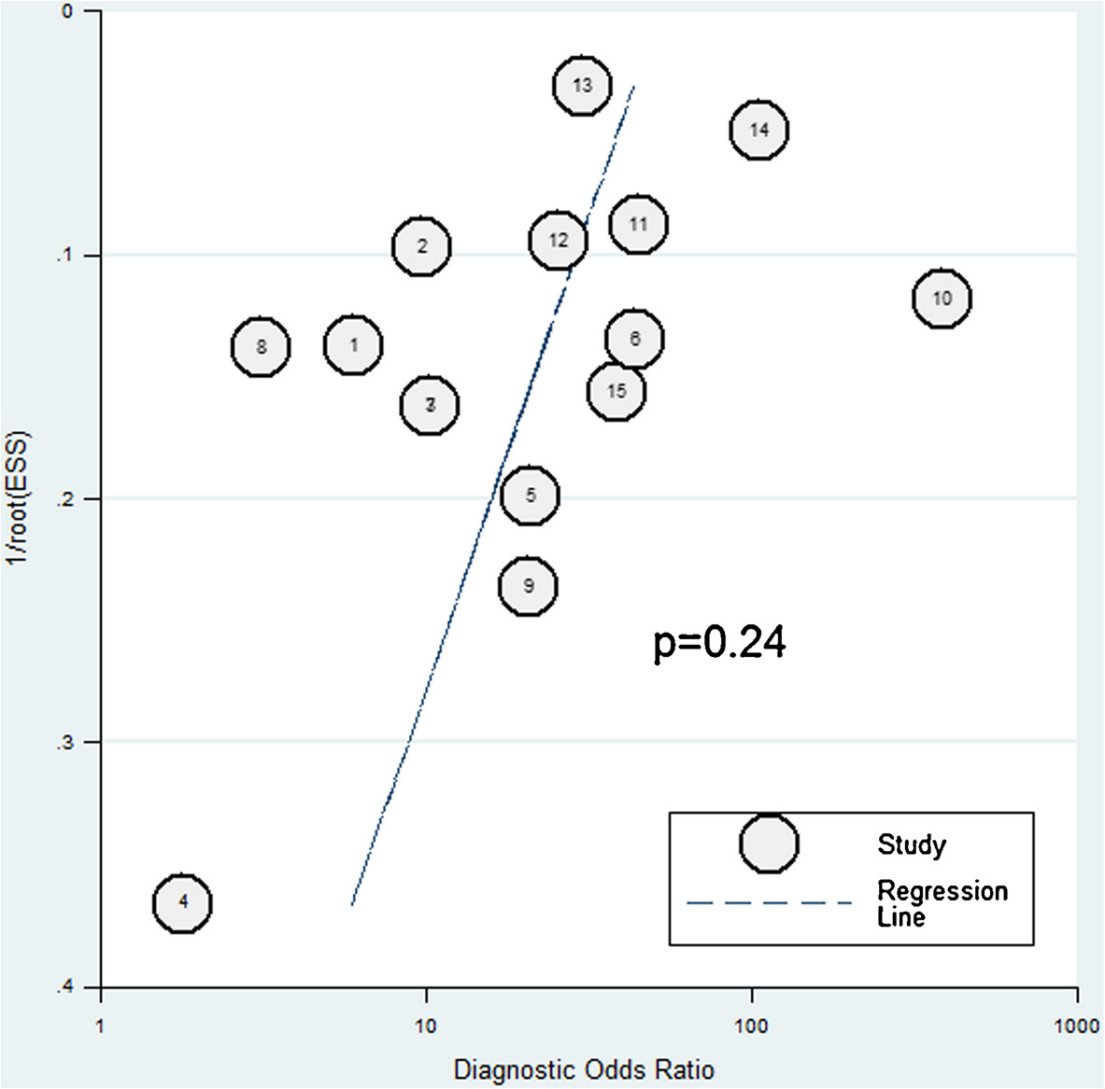
Deeks funnel plot asymmetry test of publication bias.

The Deeks funnel plot asymmetry test showed insignificant publication bias (*p*=0.24, Figure 6).

The post hoc sensitive analysis, which excluded the largest studies by Eschwege et al. and De Vegt et al., demonstrated a similar result (DOR 0.91, 95% CI 0.02-47.95, p=0.955), indicating the final conclusion of our meta-analysis was not markedly affected by these studies. Another post hoc sensitive analysis excluded studies using the 1985 WHO criteria also demonstrated a result similar to the final conclusion of the meta-analysis (DOR 0.53, 95% CI 0.01-19.36, p=0.687).

## Discussion

The role of the OGTT in the diagnosis of diabetes in the general population has been the subject of debate for decades due to the test’s poor reproducibility, which is mainly caused by random variants in glucose metabolism. Thus, it is necessary to take the reproducibility of the OGTT into consideration when evaluating the use of the test in ACS. To the best of our knowledge, this is the first meta-analysis designed to evaluate the accuracy of the OGTT in ACS and to compare it with that in non-ACS cases. In patients with ACS, OGTT has a slightly lower SPE in diagnosing diabetes compared with those without ACS, while the SEN values are comparable. A lower SPE (true negative rate) indicates that performing an OGTT in patients with ACS will result in a higher proportion of false positive results. According to the result of the subgroup analysis, less than one in every 10 ACS patients diagnosed with diabetes using an OGTT before discharge will have a different result at the follow-up OGTT resulting in a change in diagnosis. After adjusting the interval between repeated tests and age in the multiple meta-regression model, performing an OGTT in ACS patients was not associated with lower diagnostic accuracy compared with non-ACS patients. This means that it is reasonable to perform an OGTT to screen for diabetes in patients with ACS before discharge.

It is believed that aging is associated with function of the pancreatic β cells, which may affect the accuracy of OGTT. In the subgroup analysis, advanced age (≥60 years) was associated with lower SPE, while the SEN is similar when compared with the younger age (< 60 years) group. The younger patients, with well preserved function of the pancreatic β cells, maintained their plasma glucose in normal range in the setting of stress and only those with previous unknown DM could be identified during ACS. On the other hand, due to compromised function of the pancreatic β cells, the patients of advanced age were unable to maintain the plasma glucose level during the stress and the plasma glucose returned to the normal range only after the stress was eliminated, which made the OGTT less accurate in patients of advanced age enduring ACS. However, the effect of age on the accuracy of OGTT during ACS is not observed when using the meta-regression model.

Due to the poor reproducibility of the OGTT, it may be reasonable to repeat the test after discharge, which is also recommended in the WHO 1999 criteria [13]. Although the optimal time to repeat an OGTT is still unknown, OGTT could be repeated in 3 months after hospital discharge if necessary, given most of the studies repeated the OGTT 3 months after discharge.

Hyperglycemia during ACS was thought to be “stress hyperglycemia”, which develops due to a highly complex interplay between hormones (such as catecholamines, growth hormones, and cortisol) and cytokines, ultimately leading to excessive hepatic glucose production and insulin resistance [32]. A study by Choi et al. showed that acute myocardial infarction (MI) patients with IGT or diabetes exhibited higher levels of high-sensitivity C-reactive protein and interleukin-6 levels compared with acute MI patients with normal glucose tolerance or well-controlled diabetes, indicating that glycol metabolism in acute MI is associated with acute stress and inflammation [26]. However, this finding was not supported by the other studies included in this meta-analysis. Neither C-reactive protein nor the extent of myocardial damage was found to be related to hyperglycemia in ACS patients [21,23,24]. Thus, the evidence of stress hyperglycemia in ACS is still equivocal.

It is well-known that in-hospital hyperglycemia is associated with both the short-term and long-term prognosis in AMI patients [33,34]. However, either hyperglycemia on admission, fasting plasma glucose or HbA1c, in non-diabetic patients with AMI are not sensitive enough to uncover previously undiagnosed abnormal glucose tolerance or diabetes [35-37]. Furthermore, previous studies have indicated that the 2-h post challenge plasma glucose level was a significant predictor of cardiovascular events in patients with previous MI [38] and the prognosis of AMI patients with a new diagnosis of diabetes or impaired glucose tolerance defined by an OGTT during hospitalization was significantly worse than that of patients with impaired fasting glucose and normal glucose regulation [39-41]. Individuals who had normoglycemia, but whose 2-hour plasma glucose concentrations did not return to the FPG levels during an OGTT have been shown to have increased risk of cardiovascular diseases [42]. Thus, early performance of OGTT during the AMI hospitalization may provide an opportunity to detect the high risk population and establish the undiagnosed diabetes or impaired glucose tolerance. A recent study has shown that discontinuation of anti-hyperglycemic therapy during AMI hospitalization is common and associated with higher mortality rates after discharge in older patients [43]. Early diagnosis of diabetes during AMI hospitalization may be vital to improve compliance with life style changes or anti-hyperglycemic therapy.

Studies of repeated OGTT performed only in patients with abnormal or normal OGTT results in the first test were excluded from this meta-analysis, since these studies may lead to underestimation or overestimation of the accuracy of OGTT. Studies with intended medical intervention between the two OGTTs were also excluded, because several interventions such as exercise and medication have been shown to be associated with improved glucose tolerance.

The use of the hemoglobin A1C test to diagnose DM, with a threshold of 6.5%, had been recommended by ADA 2010 criteria due to its stability and convenience [44]. Recently, Ramachandran et al. divided newly diagnosed DM patients with ACS into two groups based on A1C level: undiagnosed preexisting diabetes was considered possible if the hemoglobin A1C values were ≥6.0%, and stress hyperglycemia was considered if the hemoglobin A1C values were <6.0%. Surprisingly, after 3 months, all of the undiagnosed preexisting diabetes patients remained diabetic, while only 16.7% of the stress hyperglycemia patients remained diabetic [45]. Thus, new consensus statements on the care of the hyperglycemia in ACS patients have recommended its use during hospitalization with an OGTT [46].

The studies have several limitations. First, even though the analysis identified statistically insignificant publication bias, all of the articles that fulfilled the inclusion criteria were not included because some authors were unwilling to offer the original data and there may be data relevant to this topic which have never been published. Second, these findings are based on an indirect comparison of two groups of participants, and no comparison of the reproducibility of the OGTT between ACS and non-ACS patients was available in a single study. Finally, this is a meta-analysis of study level, instead of individual level, which makes it impossible for us to determine the optimal timing for OGTT during hospitalization.

## Conclusion

Based on the meta-analysis, performing an OGTT in ACS has similar diagnostic accuracy with that in non-ACS cases. It is reasonable to use the OGTT to screen for diabetes during the hospital stay of ACS patients. Further studies should focus on the optimal timing of OGTT during ACS hospitalization.

## Abbreviations

OGTT: Oral glucose tolerance test; DM: Diabetes mellitus; ACS: Acute coronary syndrome; SPE: Sensitivity; SPE: Specificity; PLR: Positive likelihood ratio; NLR: Negative likelihood ratio; DOR: Diagnostic odds ratio; CI: Confidence interval; NICE: National Institute for Health and Clinical Excellence; WHO: World Health Organization; ADA: American Diabetes Association; ROC: Receiver operating characteristic; AUC: Area under the summary ROC curve; MI: Myocardial infarction.

## Competing interests

The authors declare that they have no competing interests.

## Authors’ contributions

YY participated in study design, literature search, data analysis, interpretation, and writing. HX participated in study design, literature search, data analysis, interpretation, and writing. XZ participated in study design, literature search, data analysis, interpretation, and writing. SZ conceived of the study, data analysis, data interpretation, and writing. All authors read and approved the final manuscript.

## Supplementary Material

Additional file 1 It includes the details of the search strategy and the assessment of study quality (risk of bias). Click here for file
