# Supplementary material for: The oral glucose tolerance test for the diagnosis of diabetes mellitus in patients during acute coronary syndrome hospitalization: a meta-analysis of diagnostic test accuracy
Source: Cardiovasc Diabetol. 2012 Dec 27;11:155. doi: 10.1186/1475-2840-11-155 (PMC3534587; doi:10.1186/1475-2840-11-155)
Supplement: Additional file 1 — It includes the details of the search strategy and the assessment of study quality (risk of bias). [file 1475-2840-11-155-S1.pdf]

SUPPLEMENTAL MATERIAL

Search strategy for systemic review

Database: MEDLINE and EMBASE (through EMBASE.com)

Date of search: 29 March 2012

Search strategy:

#1. 'oral glucose tolerance test'/exp OR 'oral glucose tolerance test'

#2. 'ogtt'/exp OR 'ogtt'

#3. 'glucose tolerance test'/exp OR 'glucose tolerance test'

#4. 'glucose tolerance'

#5. 'reproductivity'/exp OR reproductivity

#6. 'reproducible'

#7. 'reliability'/exp OR reliability

#8. 'reliable'

#9. 'variability'

#10. 'variation'

#11. 'variable'

#12. 'acute coronary syndrome'/exp OR 'acute coronary syndrome'

#13. 'heart infarction'/exp OR 'heart infarction'

#14. 'myocardial infarction'

#15. #1 OR #2 OR #3 OR #4

#16. #5 OR #6 OR #7 OR #8 OR #9 OR #10 OR#11

#17. #12 OR #13 OR #14

#18. #16 AND #17

#19. #18 AND (1985:py OR 1986:py OR 1987:py OR 1988:py OR 1989:py OR 1990:py OR 1991:py OR 1992:py OR 1993:py OR 1994:py OR 1995:py OR 1996:py OR 1997:py OR 1998:py OR 1999:py OR 2000:py OR 2001:py OR 2002:py OR 2003:py OR 2004:py OR 2005:py OR 2006:py OR 2007:py OR 2008:py OR 2009:py OR 2010:py OR 2011:py OR 2012:py)

Search results: 5521

Assessment of methodological quality

Of the 15 included studies in this review, only one abstract did not have enough information.[1] However, we have obtained access to the unpublished full-length text of the said abstract. Thus, all of the 15 studies have been under the assessment of the methodological quality.[2-15]

The items in the QUADAS tool and their interpretation were as follows:

1. *Was the spectrum of patients representative of the patients who will receive the test in practice? (Representative spectrum?)*

There are two aspects to this item: first, whether the right patient group was recruited to the study to address the review question; and second, whether the method of sampling patients for inclusion in this group was likely to yield a representative sample.

About half of the studies included the appropriate patient group to address the review question, and the method of recruitment was either consecutive or random sampling.[1, 3, 5, 7, 8, 10, 13, 14] One study did not include patients admitted during holidays.[11] The remaining studies did not specify the included criteria or the method of recruitment, which puts them at unclear risk of bias.[2, 4, 6, 9, 12, 15]

2. *Is the reference standard likely to classify the target condition correctly? (Acceptable reference standard?)*

Although the diagnostic criteria for diabetes mellitus have been changed over time, we considered all the criteria published by WHO and ADA since 1985 as appropriate reference standards.

Most of the studies mentioned the reference standard either by citing the criteria or specifying the cutoff values for diabetes. Only two studies did not mention the reference standard.[2, 5]

3. *Is the time period between the reference standard and the index test short enough to reasonably assure that the target condition did not change between the two tests? (Acceptable delay between tests?)*

Ideally, the results of the index test and the reference standard are collected on the same patients at the same time. However, the purpose of our review is to assess the reproducibility of the OGTT over time. Based on the current evidence, it is difficult to determine the optimal or reasonable interval between two tests. Thus, we considered all the studies to be at unclear risk of bias.

4. *Did the whole sample or a random selection of the sample receive verification using the intended reference standard? (Partial verification avoided?)*

Partial verification bias can occur when not all of the study patients are verified by the reference standard. Where the choice of patients for verification is not random, particularly if it is then influenced by the results of the index test, biased estimates of test performance may arise.

We have excluded studies only repeating the OGTT in patients with an abnormal result in the first OGTT. Thus, all of the patients with ACS who received the index test (the first OGTT) have gone on to receive verification of their disease status using a second OGTT. However, in most of the non-ACS studies, some of the patients who received the first OGTT did not receive verification by the second OGTT, and the

selection method for patients who would receive a second OGTT was not specified[12, 14, 15] or was only random in NGT patients.[13]

5. *Did patients receive the same reference standard irrespective of the index test result? (Differential verification avoided?)*

Although different criteria for DM have been used in the included studies, each study has used the same OGTT criteria in all patients as the reference standard. Thus, all of the included studies are at low risk of differential verification bias.

6. *Was the reference standard independent of the index test (i.e., the index test did not form part of the reference standard)? (Incorporation avoided?)*

In our review, we have used two separate OGTT as the index test and the reference standard. The index test did not form part of the reference standard. Thus, all the included studies are at low risk of incorporation bias.

7. *Were the reference standard results interpreted without knowledge of the results of the index test? (Reference standard results blinded?)*

Only one study specified that the reference standard (the second OGTT) was interpreted without knowledge of the index test (the first OGTT) results.[14] The remaining studies did not mention the blinding detail; thus, we considered them to be at high risk of diagnostic review bias.

8. *Were the index test results interpreted without knowledge of the results of the reference standard? (Index test results blinded?)*

Since we only included perspective cohort studies, the index test (the first OGTT) was performed before the reference standard (the second OGTT) was applied, and the

test results were interpreted without knowledge of the reference standard. Thus, all the included studies are at low risk of test review bias.

9. *Were the clinical data available when the test results were interpreted the same as would be available when the test is used in practice? (Relevant clinical information?)*

For some index tests, the availability or absence of relevant patient information (such as age, gender, presence and severity of symptoms, and other test results) when the test is undertaken may affect its performance. However, we used an objective measurement (blood glucose level), which is unaltered by external information. Thus, an unbiased estimate of test accuracy may be obtained by interpreting the results in isolation from other clinical information.

10. *Were uninterpretable/intermediate test results reported? (Uninterpretable results reported?*

In all the included studies, the number of results reported agreed with the number of patients recruited, indicating no uninterpretable test results.

11. *Were withdrawals from the study explained? (Withdrawals explained?)*

Most of the studies are at low risk of this bias, with six studies reporting no withdrawal[1, 3-5, 9, 14, 15] and others reporting withdrawals with explanation.[6-8, 10, 11] Three studies were considered to be at high risk of bias because of withdrawal reports without explanation.[2, 12, 13]

References:

1. Wei F. The clinical analysis of glycometabolic disturbance in patients with acute coronary syndrome. Int J Cardiol 2011;152:S112. (abstract)
2. Ilany J, Michael L, Cohen O, Matetzky S, Gorfine M, Hod H, Karasik A. Glucose homeostasis abnormalities assessed by an OGTT in coronary artery disease patients during admission and follow-up at ambulation. Exp Clin Endocrinol Diabetes 2011;119:463-6.
3. Bronisz A, Kozinski M, Magielski P, Fabiszak T, Gierach J, Swiatkiewicz I, et al. Value of oral glucose tolerance test in the acute phase of myocardial infarction. Cardiovasc Diabetol 2011;10:21.
4. Jimenez-Navarro MF, Garcia-Pinilla JM, Garrido-Sanchez L, Alonso-Briales JH, Perez-Cabeza A, Ortiz-Garcia C, et al. Poor reproducibility of the oral glucose tolerance test in the diagnosis of diabetes during percutaneous coronary intervention. Int J Cardiol 2010;142:245-9.
5. Lewczuk A, Hirnle T, Sobkowicz B, Sawicki R, Tomaszuk-Kazberuk A, Knapp M, et al. Glucose metabolism abnormalities after acute myocardial infarction in patients with one-vessel coronary artery disease, treated with primary percutaneous coronary intervention-1-year follow-up. Przeglad Kardiodiabetologinczny 2009;4:3-10.
6. Knudsen EC, Seljeflot I, Abdelnoor M, Eritsland J, Mangschau A, Arnesen H, Andersen GO. Abnormal glucose regulation in patients with acute ST-elevation myocardial infarction-a cohort study on 224 patients. Cardiovasc Diabetol

2009;8:6.

7. Srinivas-Shankar U, Somauroo JD, Delduca AM, Jordan TS, Bowles SA, Rutter MK. Temporal change in glucose tolerance non-ST-elevation myocardial infarction. Diabetes Res Clin Pract 2008;82:310-6.
8. Lankisch M, Futh R, Gulker H, Lapp H, Bufe A, Haastert B, et al. Martin S, Rathmann W. Screening for undiagnosed diabetes in patients with acute myocardial infarction. Clin Res Cardiol 2008;97:753-9.
9. Liu M, Pan CY, Jin MM, Su HY, Lu JM. The reproducibility and clinical significance of oral glucose tolerance test for abnormal glucose metabolism. Zhonghua Nei Ke Za Zhi 2007;46:1007-10.
10. Choi KM, Lee KW, Kim SG, Kim NH, Park CG, Seo HS, et al. Inflammation, insulin resistance, and glucose intolerance in acute myocardial infarction patients without a previous diagnosis of diabetes mellitus. J Clin Endocrinol Metab 2005;90:175-80.
11. Tenerz A, Norhammar A, Silveira A, Hamsten A, Nilsson G, Ryden L, Malmberg K. Diabetes, insulin resistance, and the metabolic syndrome in patients with acute myocardial infarction without previously known diabetes. Diabetes Care 2003;26:2770-6.
12. Eschwege E, Charles MA, Simon D, Thibult N, Balkau B. Reproducibility of the diagnosis of diabetes over a 30-month follow-up: The paris prospective study. Diabetes care. 2001;24:1941-4.
13. De Vegt F, Dekker JM, Stehouwer CDA, Nijpels G, Bouter LM, Heine RJ. Similar

9-year mortality risks and reproducibility for the World Health Organization and American Diabetes Association glucose tolerance categories: the Hoorn Study. Diabetes Care 2000;23:40-4.

14. Ko GTC, Chan JCN, Woo J, Lau E, Yeung VTF, Chow CC, Cockram CS. The reproducibility and usefulness of the oral glucose tolerance test in screening for diabetes and other cardiovascular risk factors. Ann Clin Biochem 1998;35:62-7.
15. Farrer M, Fulcher G, Albers CJ, Neil HAW, Adams PC, Alberti KGMM. Patients undergoing coronary artery bypass graft surgery are at high risk of impaired glucose tolerance and diabetes mellitus during the first postoperative year. Metabolism. 1995;44:1016-27.
